# Preclinical study using circular RNA 17 and micro RNA 181c-5p to suppress the enzalutamide-resistant prostate cancer progression

**DOI:** 10.1038/s41419-018-1048-1

**Published:** 2019-01-15

**Authors:** Gang Wu, Yin Sun, Zhendong Xiang, Keliang Wang, Bo Liu, Guangqian Xiao, Yuanjie Niu, Denglong Wu, Chawnshang Chang

**Affiliations:** 1 Department of Urology, Tongji Hospital, Tongji University School of Medicine, 200065 Shanghai, China; 2 George Whipple Lab for Cancer Research, Departments of Pathology, Urology, and Radiation Oncology and The Wilmot Cancer Center, University of Rochester Medical Center, Rochester, NY 14642 USA; 3 Sex Hormone Research Center, China Medical University/Hospital, 404 Taichung, Taiwan

## Abstract

Androgen-deprivation therapy (ADT) with newly developed antiandrogen enzalutamide (Enz) may increase the castration-resistant prostate cancer (CRPC) patients survival an extra 4.8 months. Yet eventually most patients may fail with development of Enz resistance. While recent clinical studies indicated that the increased expression of the androgen receptor (AR) splicing variant ARv7 might have key roles for the development of Enz resistance in CRPC, its detailed mechanism, especially its linkage to the circular RNAs (circRNAs), a form of non-coding RNA, however, remains unclear. Here we found from human clinical sample survey that circRNA17 (hsa_circ_0001427) has a lower expression in higher Gleason score PCa, and results from in vitro cell lines studies also revealed the lower expression in CRPC C4–2 Enz-resistant (EnzR-C4–2) cells compared to their parental Enz-sensitive (EnzS-C4–2) cells. Mechanism dissection indicated that suppressing circRNA17 in EnzS-C4–2 cells increased ARv7 expression that might then lead to increase the Enz resistance and cell invasion. Mechanism dissection demonstrated that Enz could suppress the circRNA17 expression at the transcriptional level via suppressing transcription of its host gene PDLIM5, and circRNA17 could regulate ARv7 expression via altering the expression of miR-181c-5p that involved the direct binding of miR-181c-5p to the 3′UTR of ARv7. Preclinical study using in vivo mouse model with xenografted EnzR-CWR22Rv1 cells revealed that adding circRNA17 or miRNA-181c-5p could suppress the EnzR-CWR22Rv1 cells growth. Together, results from these preclinical studies suggest that circRNA17 may function as suppressor to alter the Enz sensitivity and cell invasion in CRPC cells via altering the miR-181c-5p/ARv7 signaling and targeting this newly identified signaling may help in the development of a better therapy to further suppress the EnzR cell growth.

## Introduction

Prostate cancer (PCa) is the most frequently diagnosed non-cutaneous cancer in men, and is the second leading cause for men of cancer-death in western countries and the sixth most common cause in the world^1,2^. PCa can be defined as a local or advanced form clinically, and the treatments include surveillance, radical local treatment, and androgen-deprivation therapy (ADT). Most advanced PCa initially respond positively to various forms of ADT, such as medical (LHRH agonist) therapy or surgical castration. However, ADT can work only for 2 to 3 years while most patients progress to castration-resistant prostate cancer (CRPC)^3^.

Increasing evidences show that alternative androgen receptor (AR) splicing variants (AR-Vs) contribute to the development of CRPC due to their general lack of the androgen-binding domain^4–6^. To date, 15 AR-Vs have been found^6^. The ARv7 is one of the most critical AR-Vs expressed in clinical specimens^7,8^. PCa patients with a higher ARv7 expression have a shorter survival than other CRPC patients^9^. Moreover, ARv7 expression in circulating tumor cells of CRPC patients is associated with resistance to both abiraterone and enzalutamide (Enz, also known as MDV3100)^8^. These findings indicate an association between ARv7 expression and a more lethal form of PCa, and also highlight the significance of ARv7 in limiting the efficacy of ADT.

Circular RNAs (circRNAs) as a non-coding form of RNA, are widely expressed in many tissues with distinct functions to influence development of several diseases including tumor progression^10,11^. Early studies indicated that circRNAs have unique properties to allow rolling circle amplification of RNA, to rearrange the order of genomic information, and to constrain RNA folding^12^. More recently, it was found that circRNA with intron sequence can regulate transcription^13^ while RNA in circular form can also encode peptides^14–16^. Due to the circular nature of the RNAs, which endows their resistance to exonucleases, they are generally more stable and have been found to function as miRNA sponges or as miRNA reservoirs to regulate miRNA availability for breast or colorectal tumor progression^17^.

In order to explore the role of circRNAs in CRPC, we examined the expression of 21 circRNAs that potentially can bind to miRNAs that can target ARv7, and found that circRNA17 (hsa_circ_0001427) might bind to the miR-181c-5p to affect the expression of ARv7 to impact the PCa cell Enz resistance and cell invasion (Table 1).

**Table 1:** miRNAs binding to circRNA17

hsa-miR-186-5p	hsa-miR-320a
hsa-miR-1	hsa-miR-320b
hsa-miR-138-5p	hsa-miR-320c
hsa-miR-181a-5p	hsa-miR-320d
hsa-miR-181b-5p	hsa-miR-370-3p
hsa-miR-181c-5p	hsa-miR-4262
hsa-miR-181d-5p	hsa-miR-4429
hsa-miR-206	hsa-miR-494-3p
hsa-miR-27a-3p	hsa-miR-613
hsa-miR-27b-3p	

## Materials and methods

### Clinical tissues

Clinical samples of BPH and PCa were obtained from Department of Urology, Tongji Hospital, Tongji University School of Medicine, Shanghai, China; all samples were collected for research study. The written informed consent of the patients were obtained and approved by the local Medical Ethics Committee of the Tongji Hospital, Tongji University School of Medicine, China.

### Reagents and materials

ARv7 antibodies were purchased from Abcam and GAPDH (6c5) antibodies were purchased from Santa Cruz Biotechnology. Anti mouse/rabbit second antibody for Western Blot and Lipofectamine 3000 transfection reagent were purchased from Invitrogen.

### Cell culture and transfection

The human PCa cell lines, C4–2, CWR22Rv1, and 293T cell were originally purchased from American Type Culture Collection (ATCC, Manassas, VA). RPMI 1640 and DMEM were used to culture these PCa cells and 293T cell, respectively. All PCa cells were cultured in RPMI 1640 media supplemented with 10% FBS, penicillin (25 U/ml) and streptomycin (25 mg/ml) in the humidified 5% CO_2_ environment at 37 °C.

### Generation of Enz-resistant cell lines

C4–2 cells were cultured in androgen-deprived medium, then gradually increasing Enz concentration from 10, 20, 30, and then to 40 µM. C4–2 cells were treated with these Enz concentration gradient for 20 days for each concentration. After generation, cells were maintained with 10 µM Enz.

### CircRNA17 overexpression plasmid construction

The pWPI-circularRNA17 was constructed by PCR using circRNA17 forward/reverse primers to amplify the circRNA locus, which include 1 kb upstream and 200 bp downstream to the nonlinear splice sites. The PCR fragment was inserted into pWPI. An ~800-bp DNA stretch upstream was amplified by circRNA17 forward/reverse primers and inserted downstream in the reverse orientation. Furthermore, the circRNA17 exon amplified by using circRNA17-fs forward/reverse primers was inserted into pWPI to generate pWPI-circRNA17.

### Lentiviral expression plasmid construction and virus production

The pLVTHM-sh-circRNA17/pWPI-circRNA17/pWPI-AR/pLKO.1-shAR/pLKO.1-shARv7/pLVTHM-miR-181c-5p, the psAX2 packaging plasmid, and pMD2G envelope plasmid, were then transfected into HEK-293 cells using through the standard calcium phosphate transfection method. The lentivirus soups were collected after 48 h transfection and frozen in −80 °C for later use.

### Tests of RNase R resistance

Cell total RNAs were isolated by TRIZOL followed by PureLink purification of the aqueous phase (Life Technologies). One microgram of total RNA was treated with 0 U enzyme (mock treatment) or 20 U of RNase R (Epicenter) in 1 × RNase R buffer in a 10 μl reaction, 1 U/μl murine Ribonuclease Inhibitor (New England Biolabs), then incubated at 37 °C for 1 h. Then 1 μl 10 mM dNTP, 1 μl 1 mM EDTA, and 1 μl 100 M random hexamer were added in and denatured at 65 °C for 5 min, then placed on ice. Four μl 5x buffer (250 mM Tris–HCl pH 8, 125 mM KCl, 15 mM MgCl_2_), 1 μl murine Ribonuclease Inhibitor (40 U/μl), and 1 μl Superscript III (LifeTechnologies) were added; this cDNA reaction procedure was 25 °C 10 min, 50 °C 50 min, 55 °C 10 min, 85 °C 5 min, then held at 4 °C. One μl cDNA reaction was adopted as the template for qPCR.

### RNA extraction, miRNA extraction, and quantitative Real-Time PCR analysis

Total RNAs were isolated using Trizol reagent (Invitrogen, Grand Island, NY). One μg of total RNA was subjected to reverse transcription using Superscript III transcriptase (Invitrogen). Quantitative real-time PCR (qRT-PCR) was conducted using a Bio-Rad CFX96 system with SYBR green to determine the mRNA expression level of a gene of interest. Expression levels were normalized to the expression of GADPH or RPL32 RNA.

For measurement of miRNAs, briefly, 1 µg of total RNA was processed with poly A polymerase at 37 °C for 20 min, and then annealing at 65 °C for 5 min, 4 °C for 2 min after adding 50 µm RT anchor primer. The last step was cDNA synthesis at 42 °C for 60 min adding 2 μl 10 mM dNTP, 2 μl 5× RT buffer, 1 μl reverse transcriptase and ddH_2_O to total 20 μl. The qRT-pCR protocol was conducted as 95 °C 2 min, followed by 45 cycles at 95 °C 15 s, 60 °C 45 s. U6 and/or RPL32 were used as controls for normalization.

### Western blot analysis

Cells were lysed in RIPA buffer and proteins (30 µg) were separated on 8–10% SDS/PAGE gel and then transferred onto PVDF membranes (Millipore, Billerica, MA). Membranes were blocked by 5% Bovine Serum Albumin (Sigma-Aldrich, St. Louis, MO) for 1 h at room temperature and then incubated with appropriate dilutions of specific primary antibodies, and then incubated with HRP-conjugated secondary antibodies and visualized using ECL system (Thermo Fisher Scientific, Waltham, MA).

### Cell invasion assay

The invasion capability of PCa was determined using the transwell and 3D invasion assay. Briefly, cells were cultured with serum-free media into the upper chamber at 1 × 10^5^ cells per well pre-coated with Matrigel (BD Corning). The lower chambers contained DMEM with 10% FBS. After 48 h incubation, the non-invading cells and matrigel in the upper chambers were removed gently by cotton swabs and cells invaded to the bottom of the membranes were fixed by 4% paraformaldehyde and stained with 1% toluidine blue. The cell numbers were calculated from counting six random fields. Quantitation is presented as mean ± SEM of triplicate repeats.

### The three-dimensional invasion assay

The three-dimensional invasion assay was performed as described^18^. Briefly, 5 × 10^4^ cells in 1.5 ml media containing 1% matrigel were plated into the 24-well plate coated with collagen/matrigel mixture. The media were exchanged every 3 days. The spheres with protrusion were recorded as the valid invasion cell. The spheres with/without protrusion in each well were observed under the Olympus IX70 inverted microscope within 2 weeks. Each experiment was repeated twice and each experiment was done in triplicates.

### Luciferase assay

The human ARv7 3′UTRs containing wild type or mutant miRNA-responsive elements were cloned into the psiCHECK™−2 vector construct (Promega, Madison, WI) downstream of the Renilla luciferase ORF. Luciferase activity was measured by Dual-Luciferase Assay (Promega) according to the manufacturer’s manual.

### Chromatin immunoprecipitation assay (ChIP)

Cells were cross-linked with 30% formaldehyde for 10 min followed by cell collection and sonication with a predetermined power to shear chromatin to 300–700 bp DNA fragments. Cell lysates were precleared sequentially with normal rabbit IgG (sc-2027, Santa Cruz Biotechnology) and protein A-agarose. Anti-AR antibody (2 µg) was added to the cell lysates and incubated at 4 °C overnight. IgG was used as the negative control. Specific primer sets were designed to amplify a target sequence within the human AR promoter. PCR products were identified by agarose gel electrophoresis.

### Pull-down assay

The culture cells were collected and lysed in RIPA lysis buffer. The supernatant was mixed with 500 pM of anti-sense oligos supplemented with RNAase inhibitor overnight at 4 °C. The cells were mixed for 2 h at 4 °C after adding 10ul Streptavidin Agarose beads; then the streptavidin Agarose beads were incubated with the supernatant for 2 h. The complex was centrifuged at speed of 3000 rpm for 10 mins, and the beads were washed with RIPA lysis buffer for five times. The RNA was extracted using Trizol (Invitrogen) according to the manufacturer’s protocol and subjected to RT-PCR analysis.

### In vivo orthotopic implantation, tumor formation, and Enz treatment

Six-eight-weeks-old male nude mice were injected orthotopically into the anterior prostate with CWR22Rv1 cells (1 × 10^6^ suspended in 200 μl Matrigel). The naturally resistant CWR22Rv7 cells were also transduced with luciferase for IVIS monitoring. After xenograft tumors grew to about 0.2 cm^3^, animals were injected with 30 mg/kg enzalutamide for 5 weeks before animal was killed. The mice were monitored with IVIS weekly in the process. All the animal experiments were performed in accordance with the guidelines of the University of Rochester Medical Center Animal Care and Use Committee for animal experiments.

### H&E and immunohistochemical (IHC) staining

Mouse prostate tissues were fixed in 10% (v/v) formaldehyde in PBS, embedded in paraffin, and cut into 5 μm sections. Prostate sections were deparaffinized in xylene solution and rehydrated using gradient ethanol concentrations. EDTA was used for antigen retrieval at 98 °C for 20 mins. Slides were incubated with H_2_O_2_ to block the endogenous peroxidase and then incubated with the primary antibody at 4 °C overnight; then the slides were incubated for 45 mins with biotin-conjugated secondary antibody, after rinsing with Tris buffered saline. Freshly prepared DAB (Zymed, South San Francisco, CA) was used as substrate to detect HRP. Finally, slides were counter-stained with hematoxylin and mounted with aqueous mounting media. Positive cells were calculated as the number of immunopositive cells × 100% divided by total number of cells/field in 10 random fields at ×400 magnification.

### Statistical analysis

Data were expressed as mean ± SEM from at least three independent experiments. Two-tailed Student’s *t*-test was used for differences in mean values between two groups. The mean values of more than two groups were compared with one way analysis of variance. *P* ≤ 0.05 was considered statistically significant.

## Results

### The circRNA17 regulates the expression of ARv7 in CRPC C4–2 cells

Non-coding RNAs have been increasingly recognized as being critical for all the biological processes examined^10,11^. The circRNAs can exert their functions mainly as regulators of miRNA availability, however, their influences on the expression of ARv7 and their roles on the development of Enz resistance remain unclear.

**Fig. 1:**
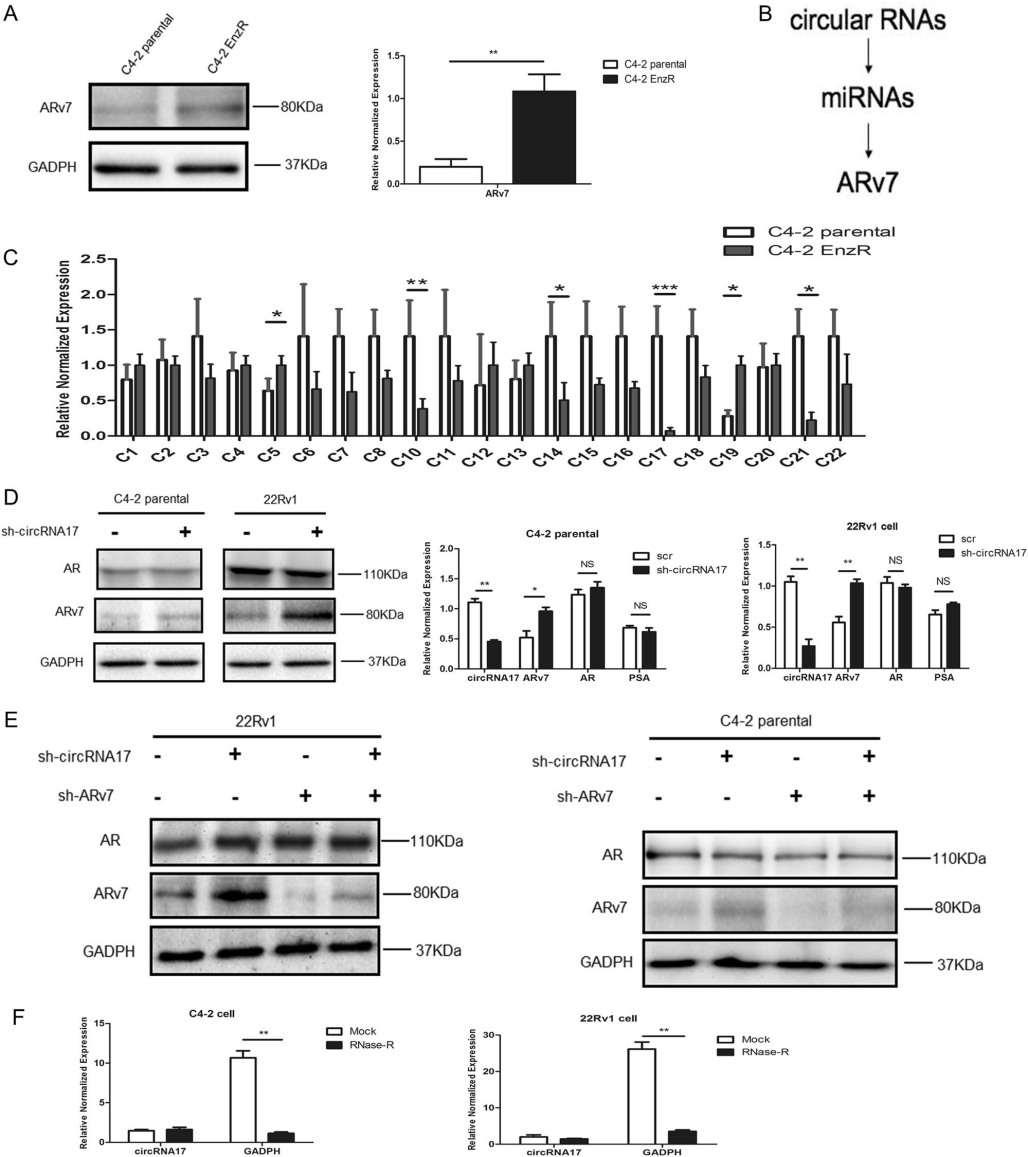
CircRNA17 regulates expression of ARv7 in C4–2 cell lines. **a** ARv7 expression at the protein (left panel) and transcript level (right panel) in C4–2 parental cells and C4–2 EnzR cells. **b** Selection of circular RNAs that can potentially regulate miRNAs that target ARv7. **c** Semi-quantitative PCR analysis of 21 circRNAs in C4–2 parental cell and C4–2 EnzR cells. **d** Knocking down circRNA17 results in increase of ARv7 both at the protein (left panels) and transcript level (right panels). **e** Knocking down circRNA17 results in increase of ARv7, which can be decreased by knocking down ARv7 in both CWR22Rv1 (22RV1) and C4–2 parental cells. **f** RNase R assay to determine the sensitivity of circRNA17 by RNase R digestion in C4–2 (left panel) and CWR22Rv1 (right panel) cells. *p < 0.05, **p < 0.01, ***p < 0.001, NS = Not significant

We first confirmed the potential linkage of ARv7 expression to the Enz resistance in vitro by showing its increased expression in Enz-resistant C4–2 (EnzR-C4–2) cells that were developed using a prolonged treatment with 10 µM Enz (Fig. 1a), which is continued throughout all experiments. We hypothesized that there might be circRNAs whose expression would be correlated with ARv7 expression and Enz resistance. In a candidate approach, we identified miRNAs that can target ARv7 through their unique 3′UTRs as well as those that were in a lower expression in recurrent PCa^18^ while also bioinformatically predicted to bind to 21 circRNAs (Fig. 1b). We then examined ARv7 linkage to the differential expression of these 21 circRNAs during development of Enz resistance as compared between EnzR-C4–2 cells and the parental Enz-sensitive (EnzS-C4–2) cells (Fig. 1c) that were grown for the same long-term time as experimental controls for the EnzR-C4–2 cells.

We then examined the consequence of reducing the circRNAs on the expression of ARv7 in EnzS-C4–2 parental cells. The results indicated that knocking down circRNA17 with shRNA in EnzS-C4–2 parental cells resulted in an increase of ARv7 at both mRNA and protein levels, indicating that circRNA17 was capable of regulating ARv7 expression (Fig. 1d), likely through regulating miRNA stability/level rather than sponging the target miRNAs to regulate ARv7, as this circRNA17 is expressed higher in EnzS-C4–2 parental cells than in EnzR-C4–2 cells. In addition, reduction of this circRNA in both EnzS-C4–2 and CWR22Rv1 cells resulted in an increase of ARv7, which can be reversed by a specific knockdown of ARv7 (Fig. 1e). Furthermore, RNase R digestion assays demonstrated that circRNA17 is indeed in the circular form as it is resistant to the RNase R digestion in both EnzS-C4–2 and CWR22Rv1 cells (Fig. 1f).

Together, results from Fig. 1a–f using multiple CRPC cells suggest that ARv7 contributes to the development of Enz resistance in CRPC cells and circRNA17 can regulate ARv7 expression.

### Targeting circRNA17 alters ARv7-induced Enz resistance and cell invasion in the CRPC cells

**Fig. 2:**
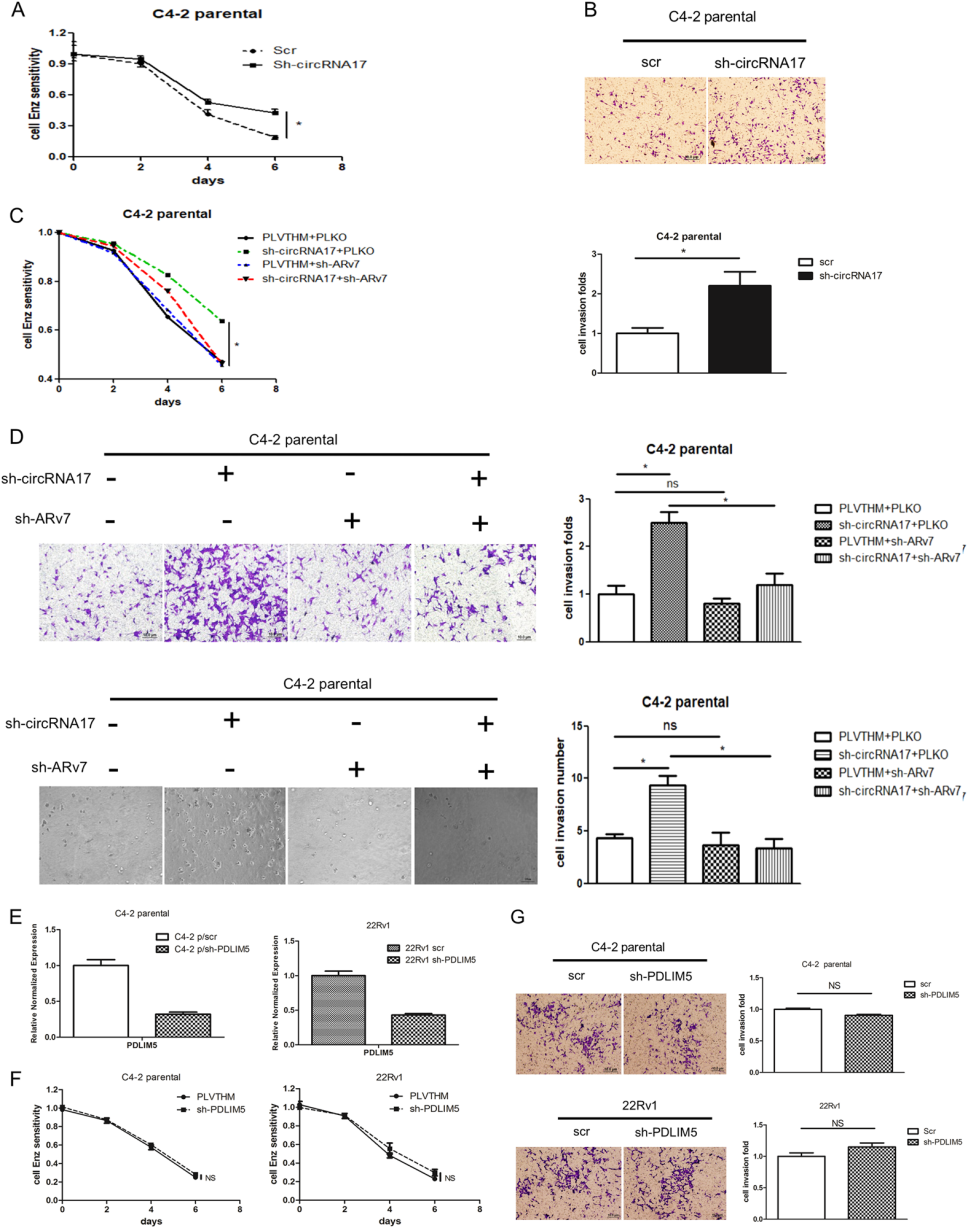
The circRNA17 may influence PCa proliferation and cell invasion via altering the ARv7. **a** Knocking down circRNA17 in C4–2 parental cell results in decrease of Enz sensitivity. MTT assay was used to measure the cell proliferation in response to 10 µM Enz treatment in the C4–2 parental cells with a knockdown of circRNA17 (sh-circRNA17) or vector control (PLVTHM). **b** Knocking down circRNA17 in C4–2 parental cells increased cell invasion. Boyden chamber assay was used to measure the cell invasion in C4–2 parental cells with sh-circRNA17 or PLVTHM control cells. Quantitation is in lower panel. Scale bar, 10 µm. **c** The decrease of Enz sensitivity from knocking down circRNA17 can be reversed by a simultaneous knockdown of ARv7. MTT assay was performed as in **a** in cells with the indicated expression of various shRNA constructs. **d** The increase of cell invasion in transwell assay (upper) and 3D invasion (lower) assay as a result of sh-circRNA17 can be reversed by a simultaneous knockdown of ARv7 in C4–2 parental cells. Boyden chamber assay was performed in as in **b** in cells with the indicated shRNA constructs. Scale bar, 10 µm. **e** Knocking down PDLIM5 linear sequence outside the circRNA17 region in C4–2 and CWR22Rv1 (22Rv1) cells. **f**, **g** Knocking down PDLIM5 host gene linear sequence did not alter in C4–2 and 22Rv1 cells Enz sensitivity (**f**) and transwell invasion (**g**). Scale bar, 10 µm. For **d** and **g**, quantitations are at the right. *p < 0.05, **p < 0.01 and ns (or NS) = no statistical differences

Next, to study the biological consequences of knocking down circRNA17 in the CRPC cells, we subjected the parental EnzS-C4–2 cells (with/without knocked-down circRNA17) to 10 µM Enz treatment. The results revealed that knocking down of circRNA17, and therefore increasing the ARv7 expression, might increase C4–2 parental cells’ resistance towards Enz treatment compared to the vector control cells (Fig. 2a), as well as increased cell invasion in EnzS-C4–2 parental cells using matrigel-chamber assay (Fig. 2b).

Importantly, we further found that increased Enz resistance in C4–2 parental cells with knocked-down circRNA17 could be reversed partially via knocking down ARv7 with ARv7-shRNA (Fig. 2c). In a concordant fashion, increased cell invasion via knocking down circRNA17 was also reversed partially after knocking down ARv7 with both chamber assay and 3D invasion assay (Fig. 2d). To exclude the off-target possibility of sh-circ17 on its linear host gene mRNA, we used shRNA construct that targeted the sequence outside the circRNA region and found that knocking down the linear host gene RNA sequence did not affect Enz sensitivity and transwell invasion in both C4–2 and 22Rv1 cells (Fig. 2e–g), further attesting the specificity of sh-circ17 on circ17 for its functional consequences.

Together, results from Fig. 2a–g suggest that targeting circRNA17 can alter the ARv7 expression to alter the Enz resistance and Enz-increased cell invasion in the CRPC cells.

### Mechanism dissection of how Enz can suppress the circRNA17 expression: via altering the transcriptional regulation of the circRNA17 host gene PDLIM5

**Fig. 3:**
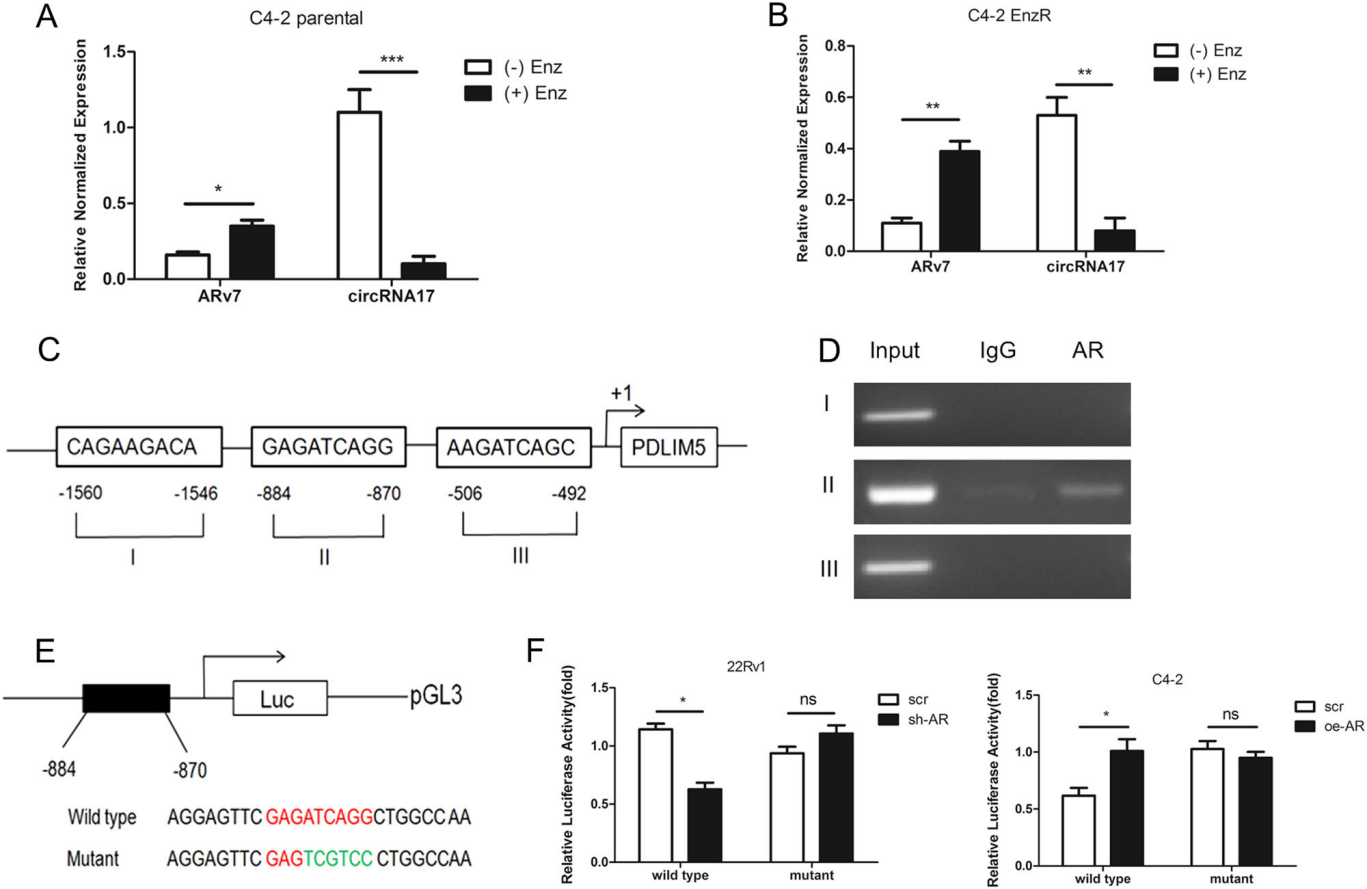
Enz can regulate circRNA17 expression in an AR-dependent manner. **a** Enz can repress circRNA17 expression in C4–2 parental cell with a concomitant increase of ARv7 at the transcript level. C4–2 parental cells were treated with 10 µM Enz for 48 h followed by the RNA isolation and measurement of circRNA17 and ARv7 with RT-qPCR. **b** Withdrawal of Enz in C4–2 EnzR cells results in an increase of circRNA17 as well as ARv7 at the transcript level. **c** Bioinformatic analysis of putative AR-binding sites in circRNA17 host gene PDLIM5 promoter. **d** ChIP assay indicates AR binds to ARE at position 876–884 upstream of PDLIM5 transcription start site. **e**, **f** Transfection of PDLIM5 5′ promoter constructs containing wild type or mutant ARE sequence into C4–2 and CWR22RV1 (22RV1) cells and luciferase assay was applied to detect the luciferase activity. For **a**, **b**, and **f**, relative normalized expressions are shown. *p < 0.05, **p < 0.01, ***p < 0.001 and ns=no statistical differences

Although EnzR-C4–2 cells had a lower expression of circRNA17 with an increase of ARv7, it is not clear whether an acute treatment with Enz will also result in a change in circRNA17 expression and hence ARv7 expression. We therefore examined the circRNA17 expression in EnzS-C4–2 parental cells after 48 h treatment with 10 µM Enz, and results revealed that acute treatment of Enz decreased circRNA17 expression with a concomitant increase of ARv7 expression (Fig. 3a). Consistent with this, deprivation of Enz in the C4–2 EnzR cells led to an increase of circRNA17 with a decrease of ARv7 expression (Fig. 3b).

Together, results from Fig. 3a and b suggest Enz can directly suppress the circRNA17 expression to increase the expression of ARv7.

As circRNAs are usually produced as a result of transcription of the host gene, in this case, PDLIM5 for circRNA17, it is likely that Enz, through suppressing AR activity, may link to the repression of the PDLIM5 gene transcription. Indeed, through bioinformatics analysis (PROMO website), three potential androgen-response elements (AREs) were found in the 2 kb promoter region of circRNA17 host gene PDLIM5 (Fig. 3c).

We then applied the ChIP assay and results revealed that AR could bind to the ARE located on 870–884 bp upstream of the transcription start site of PDLIM5 (Fig. 3d). We also generated a reporter construct bearing the wild-type sequence and a construct with a mutation in the ARE (Fig. 3e). Results from luciferase reporter assay with the 1 kb promoter region confirmed that knocking down AR in CWR22Rv1 cells, a mimic of Enz treatment, can lead to a reduction of reporter activity of the wild-type promoter while fail to do so with the mutant reporter construct (Fig. 3f, left panel). Consistent with this, increasing AR with adding AR-cDNA in C4–2 cells increased the promoter activity of the wild-type but not the mutant reporter (Fig. 3f, right panel).

Together, results from Fig. 3a–f suggest that Enz can transcriptional suppress the circRNA17 expression via suppressing transcription of its host gene PDLIM5.

### Mechanism dissection of how circRNA17 alters the ARv7 expression in CRPC cells: via regulating the miR-181c-5p expression

Next, to dissect the molecular mechanism of how circRNA17 can alter the ARv7 expression, we focused on the miRNAs, since recent studies indicated that circRNAs might function as sponge or reservoir for various miRNAs to regulate breast or colorectal tumor progression^17^.

**Fig. 4:**
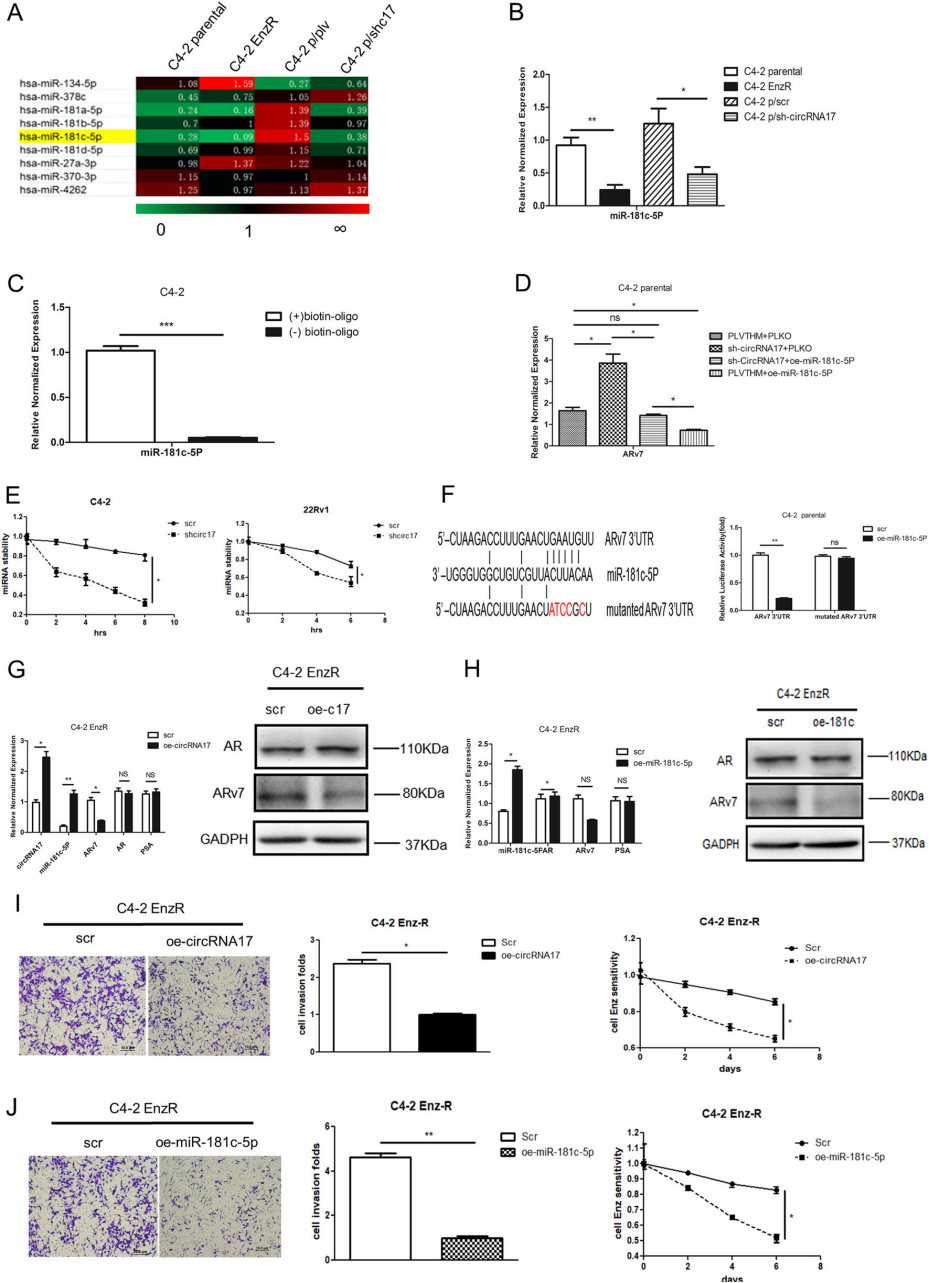

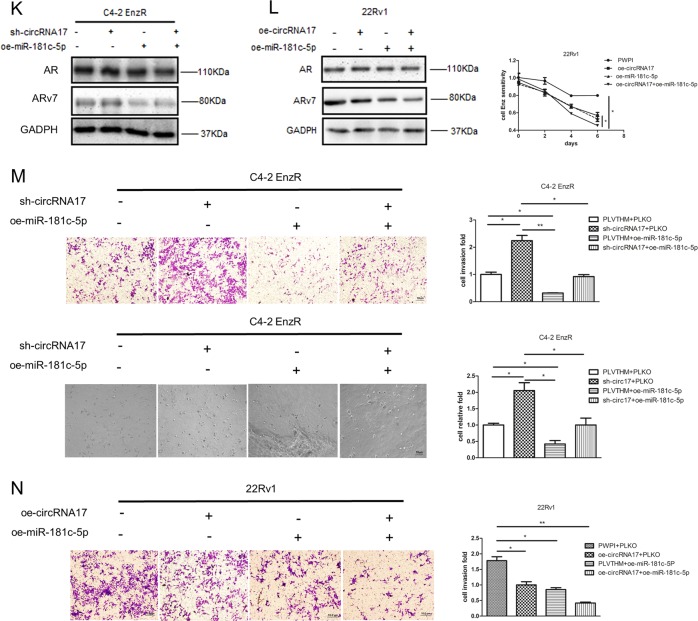
The circRNA17 regulates ARv7 via miR-181c-5p. **a** Overview of qPCR assay for 9 miRNAs that can bind to both circRNA17 and ARv7 in C4–2 parental and C4–2 EnzR cells, C4–2 parental cell, and sh-circRNA17 cell line. Nine miRNAs were significantly suppressed in both C4–2 parental and C4–2 parental with sh-circRNA17 cell lines. **b** The miR-181c-5p expression in C4–2 parental and EnzR cells as well as in C4–2 parental cells with/without knockdown of circRNA17. **c** The miR-181c-5p can physically interact with circRNA17. The biotinylated oligo that is complimentary to the junction region of circRNA17 was mixed with the C4–2 parental cell lysate followed by purification with streptavidin beads. The miR-181c-5p was measured in the pull-down complex as well as control complex without the biotinylated oligo. **d** ARv7 transcript levels in C4–2 parental cells with a knocked-down circRNA17 as well as in cells with overexpressed miR-181c-5p. **e** The miR-181c-5p stability in cells with a knockdown of circRNA17. The miR-18c-5p stability was measured with the addition of 5 µM Actinomycin D in C4–2 parental and CWR22Rv1 (22Rv1) cells with/without knocked-down circRNA17. **f** Predicted miR-181c-5p target sites in ARv7 3′UTR. The right panel indicates that a luciferase reporter assay with psiCheck2 vector carrying a wild-type 3′UTR of ARv7 can be suppressed by overexpression of miR-181c-5p while that of the mutant 3′UTR could not. **g** Overexpression of circRNA17 in C4–2 EnzR cells resulted in increase of miR-181c-5p as well as a repression of ARv7 at the transcript (left panel) and protein level (right panel). **h** Overexpressing miR-181c-5p in C4–2 EnzR cells repressed the ARv7 expression at the transcript (left panel) and protein level (right panel). **i** Overexpression of circRNA17 in C4–2 EnzR cells repressed cell invasion (left panel) as well as increased sensitivity (right panel) of Enz. MTT assay was used to measured the proliferation in response to 10 µM Enz in C4–2 EnzR cells. Scale bar, 10 µm. **j** Overexpression miR-181c-5p in C4–2 EnzR cell repressed cell invasion as well as (right panel) increased sensitivity of Enz. MTT assay was used as in **i**. Scale bar, 10 µm. **k** Overexpression of miR-181c-5p in C4–2 Enz-resistant cells resulted in suppression of ARv7 that was induced by knocking down circRNA17. **l** Overexpression of miR-181c-5p and circRNA17 in 22Rv1 cells could suppress ARv7 expression (left panel) as well as increase sensitivity of Enz (right panel). **m** Overexpression of miR-181c-5p in C4–2 EnzR cells repressed invasion in transwell assay and 3D invasion assay that was induced by knocking down circRNA17. Scale bar, 10 µm. **n** Overexpression of circRNA17 or miR-181c-5p in 22Rv1 cells could suppress cell invasion. Scale bar, 10 µm. For **f**, **m**, and **n**, quantitations are at the right. *p < 0.05, **p < 0.01, ***p < 0.001, and ns = not significant

Through bioinformatics analysis of existing databases (DIANA miRGen, MicroCosm Targets, RNA22, and RegRNA2.0) and published literature, we found circRNA17 might be able to interact with several miRNAs, which can also bind to ARv7 3′ untranslated region (UTR) (Fig. 4a). Among those potential candidate miRNAs, we focused on miR-181c-5p, since its expression was higher in C4–2 parental cells, while lower when circRNA17 was suppressed (Fig. 4b). Importantly, an early study also indicated that miR-181c-5p might have a key role in the cancer stem cell formation^19^.

We then applied the pull-down assay using the biotinylated oligo, to examine the interaction of circRNA17 with miR-181c-5p in C4–2 parental cells, and results revealed that circRNA17 could interact with miR-181c-5p (Fig. 4c).

Importantly, an interruption approach via expressing miR-181c-5p reversed the shcircRNA17-increased ARv7 transcript level, consistent with miR-181c-5p’s interaction and suppression of ARv7 mRNA (Fig. 4d).

As circRNA17 is positively correlated with the expression level of miR-181c-5p, this circRNA may not function as a sponge to sequester the miRNAs, but likely function as a reservoir or a stabilizing factor for the miR-181c-5p. Indeed, as shown in Fig. 4e, the stability of miR-181c-5p is reduced when circRNA17 is silenced, with a consequent decrease of expression level in the C4–2 parental and CWR22Rv1 cell lines.

Together, results from Fig. 4a–e suggest circRNA17 may function via regulating the miR-181c-5p expression to modulate the ARv7 expression.

To further clarify the molecular details of how circRNA17-regulated miR-181c-5p can lead to alter the ARv7 expression, we identified 2 potential miRNA-target sites that matched the seed sequence of miR-181c-5p on the 3′UTR of ARv7 transcript (Fig. 4f, left panel). We then inserted the ARv7 3′UTR into a dual-luciferase reporter that contains the predicted miR-181c-5p target site at 915–1196 nt, and a mutated version at the predicted target site. Results from the luciferase assays revealed that expressing miR-181c-5p markedly decreased luciferase activity in C4–2 parental cells transfected with wild-type ARv7 3′UTR, but not the mutant ARv7 3′UTR (Fig. 4f, right panel), suggesting that miR-181c-5p can directly modulate the ARv7 expression through binding to its 3′UTRs.

Results from Fig. 4f suggest that circRNA17-modulated miR-181c-5p can alter the ARv7 expression via directly binding to the ARv7 3′UTR.

### The circRNA17/miR-181c-5p/ARv7 signaling alters the CRPC cell invasion and Enz resistance

To further determine the biological significance of circRNA17/miR-181c-5p/ARv7 signaling, we altered the expression of these molecules and observed the responses towards Enz resistance and cell invasion. Results from these interruption approaches indicated that adding circRNA17 or miR-181c-5p decreased the ARv7 expression at both mRNA and protein levels (Fig. 4g, h). Importantly, adding circRNA17 and miR-181c-5p in EnzR-C4–2 cells also decreased cell invasion and reduced sensitivity towards Enz (Fig. 4i, j).

Similar results were also obtained when we replaced EnzR-C4–2 cells with the naturally Enz resistant CWR22Rv1 cells showing adding circRNA17 and miR-181c-5p led to decreasing the cell invasion and Enz sensitivity (Fig. 4l). In addition, the close relationship between circRNA17 and miR-181c-5p on regulating ARv7 expression was also underscored by the finding that miR-181c-5p can repress the increased ARv7 expression induced by knocking down circRNA17 in CRPC cell lines (Fig. 4k, l). Moreover, this relationship between circRNA17 and miR-181c-5p can also be functionally manifested in cell invasion in two different cell invasion assays including transwell assay and 3D invasion assay (Fig. 4m, n) with miR-181c-5p expression reversing an increase of cell invasion induced by knocking down circRNA17 in CRPC cell lines.

Together, results from Fig. 4a–n using multiple EnzR cells with different assays suggest that Enz may function via altering the circRNA17/miR-181c-5p signaling to modulate ARv7 expression that results in altering the Enz sensitivity and cell invasion in CRPC cells.

### Preclinical study using in vivo mouse model to demonstrate the functional significance of circRNA17 in altering Enz sensitivity

To demonstrate the in vivo role of circRNA17 and miR-181c-5p in a xenograft mouse model, we used CWR22Rv1 cells with stable expression of luciferase to allow the non-invasive monitoring through in vivo imaging system (IVIS) in mice and generated four groups of cells with expression of PWPI control, circRNA17, miR-181c-5p, and simultaneous expression of circRNA17 and miR-181c-5p. After being injected with the cells, all mice were treated with Enz. These groups of cells were designed to test the role of circRNA17 as a stabilizing factor/reservoir of miRNA-181c-5p as demonstrated in vitro rather than a sponge of miRNA as circRNA is generally believed to be.

**Fig. 5:**
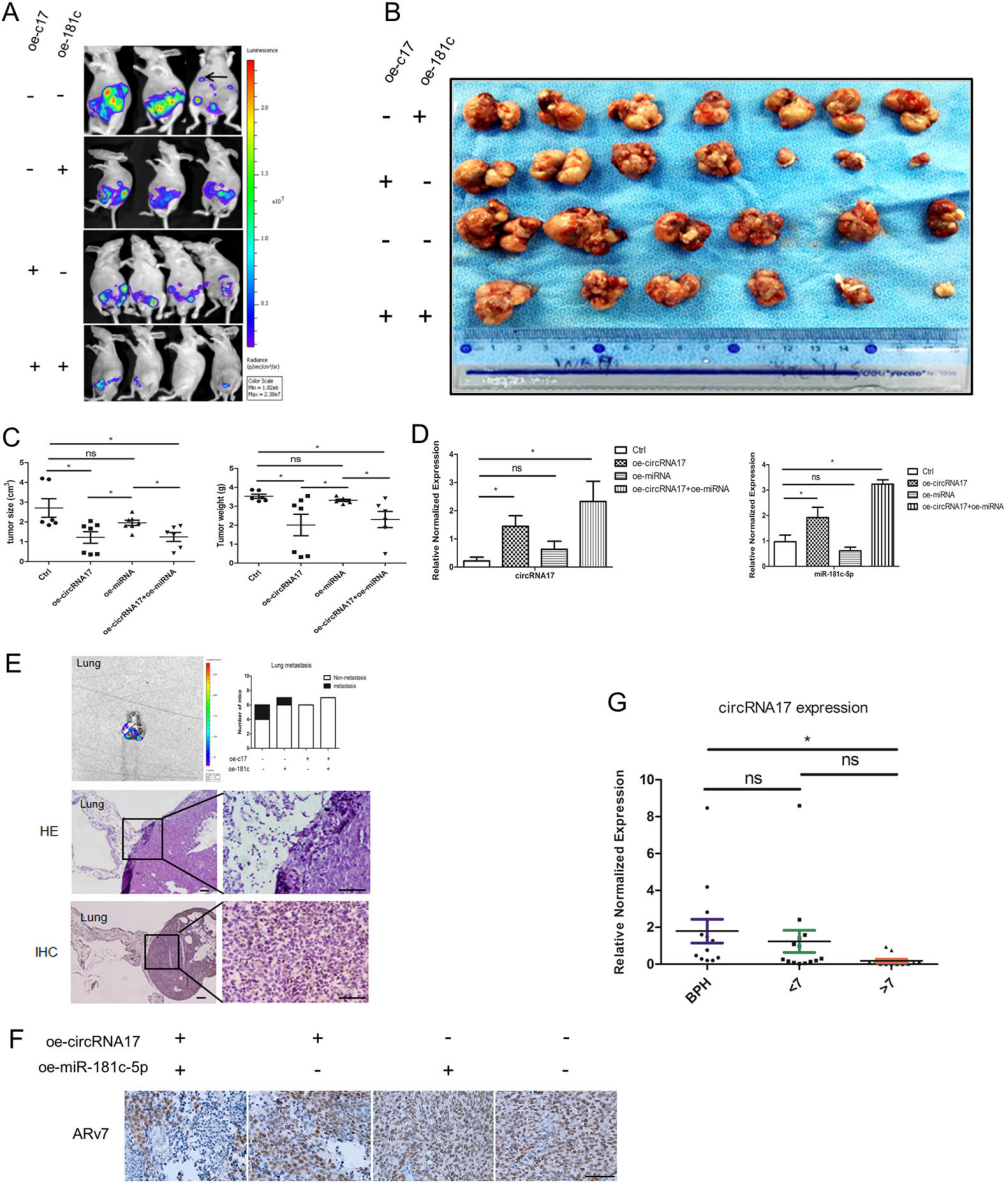
In vivo studies confirmed the role of circRNA17 and miR-181c-5p in PCa metastasis and Enz resistance. Mice with orthotopic implantation of CWR22Rv1-luc cells with overexpressed circRNA17 (oec17) show reduced PCa invasion and Enz resistance. The CWR22Rv1-luc cells (1 × 10^6^) infected with different lentiviral constructs were orthotopically implanted into the AP of nude mice. Mice were imaged weekly by IVIS. After 8 weeks implantation, the tumor/metastases growth was monitored again by IVIS analysis and mice sacrificed and tumors/ metastases removed for studies. **a** IVIS was used to determine the tumor size and metastasis. **b** Macroscopic appearance of the tumor xenografts. **c** Sizes and weights of the xenografts were tabulated. **d** The expression of circRNA17 and miR-181c-5p in the four groups of mice, including control, oe-circRNA17, oe-miRNA,and oe-circRNA17 + oe-miRNA, numbering 6, 7, 7, and 6, respectively. **e** Quantification of the lung metastases and representative bioluminescent images of lung metastases (upper panel); HE staining (middle panel) and IHC (lower panel) confirmed the tumor tissue in lung. Scale bar, 100 µm. **f** Representative images of IHC staining for ARv7. Scale bar, 100 µm. **g** The expression of circRNA17 in BPH samples (n – 13), or PCa samples with Gleason score < 7 (*n* = 14), and Gleason score > 7 (*n* = 12)

Equal numbers of cells were injected orthotopically into the anterior prostates (AP) of mice followed by treatment with Enz (30 mg/kg body weight, three times per week). The IVIS was used to monitor the PCa progression^20^. We found 2 out of 6 mice with CWR22Rv1-luc cells transfected with PWPI and 1 out of 7 mice with CWR22Rv1-luc cells transfected with overexpression of miR-181c-5p had metastatic foci. In contrast, no metastatic foci were found in mice with CWR22Rv1-luc cells transfected with circRNA17 and miR-181c-5p cells (Figs. 5a, e). These mice were killed after 8 weeks and the tumors were collected for sizes and weights (Fig. 5b, c). Furthermore, the expression of circRNA17 and miR-181c-5p and metastasis were measured in four groups (Fig. 5d, e). The mean tumor size and tumor weight in PWPI group were the highest in these four groups, while the circRNA17 plus miRNA-181c-5p was the lowest by tumor size measurement as well as metastatic foci (Fig. 5a–c). Significantly, the overexpression of miRNA-181c-5p alone did not achieve higher expression than the vector control while simultaneous overexpression of miRNA-181c-5p together with circRNA17 did, suggesting that circRNA17 can stabilize miR-181c-5p expression in vivo (Fig. 5d), consistent with the tumor weight and volume measurements (Fig. 5c) Importantly, results from ARv7 IHC staining in the xenograft tumor samples matched well with the miR-181c-5p expression as well as findings from in vitro cell line studies (Fig. 5f). These data from the animal studies confirmed that circRNA17 serves as a reservoir, rather than a sponge of miRNA-181c-5p as circRNA17 enhanced miR-181c-5p function to suppress the ARv7 expression and tumor progression.

### Human clinical survey for the functional significance of circRNA17 expression

To further demonstrate the functional significance of circRNA17 expression in human clinical samples, we compared its expression in benign prostatic hyperplasia and different stages of PCa progression. As shown in Fig. 5g, circRNA17 was expressed at the lowest level in the PCa with gleason score above 7, consistent with the role of circRNA17 as a tumor suppressor, likely through stabilizing the expression of miRNA-181c-5p among other miRNAs.

## Discussion

Advanced PCa is an aggressive disease, and an estimated 1,100,000 new cases of PCa were diagnosed worldwide in 2012^2^. ADT remains the primary therapy for advanced PCa patients, however, most patients relapse and invariably progress to CRPC within a median of 18–24 months^21^. Emerging evidences show that AR splicing variants may promote the PCa progression even at the stage of CRPC^7,22^. To date, more than 20 AR splice variants have been identified and ARv7 is one of the most abundant and best characterized variant^8,23^.

In an evaluation of ARv7 at the mRNA level, it was found to be on average 20-fold higher in CRPC compared with hormone-naive PCa (*p* < 0.001) and there was elevated expression of ARv7-predicted biochemical recurrence after radical prostatectomy among the hormone-naive PCa patients^24^. Results from a large study containing 429 human PCa specimens showed that ARv7 was significantly upregulated during PCa progression and expression of ARv7 in primary PCa was predictable for survival after radical prostatectomy, with a shorter time to biochemical recurrence in individuals with elevated levels (*p* < 0.001)^7^. Our recent study also revealed that those PCa patients who have higher expression of ARv7 may develop to EnzRPCa and ADT with Enz failed to get pleasing results^25^.

CircRNAs are non-coding RNAs with high conservation and abundance and form closed continuous loops from exons or introns by back splicing or lariat formation. Compared with linear RNAs, circRNAs are more stable due to their higher resistance to nuclease activity, and have a higher expression in some tissues and developmental stages^26,27^. CircRNAs have been found to function as a novel class of biomarkers and natural miRNA sponges^28–30^ and are also called competing endogenous RNAs (ceRNAs) in diverse species.

Lukiw WJ demonstrated that a circRNA for miRNA-7 can function as a kind of endogenous, competing, anti-complementary miRNA “sponge” to absorb and degrade miRNA-7, thus affect the Alzheimer’s disease progression^31^. CircRNAs are more effective than typical linear miRNA sponges and miRNA-inhibitors in de-repressing miRNA targets thus showing superior anti-cancer activities compared to the linear sponges in malignant mel-an-o-ma cell lines^32^. More recently a circRNA (ITCH) was found to regulate the development of esophageal squamous cell carcinoma via the Wnt/β-catenin pathway^33^.

We provided the clear demonstration that a circRNA can also serve as a reservoir or a stabilizing factor for miRNA expression, opposite to the sponge function to suppress miRNA. This novel function of circRNA was demonstrated in vitro through cell line studies showing the congruent function of circRNA17 and miRNA-181c-5p in suppressing ARv7 expression, as well as in vivo with animal studies showing expression of both ncRNAs resulted in the most significant reduction of ARv7 expression and tumor growth and metastasis in response to Enz treatment. Mechanistically, we can speculate the binding of miR-181c-5p with circRNA17 might enhance the miR-181c-5p stability by repressing its degradation through nucleases such as Tudor-SN endonuclease^34^. In addition, this stabilizing effect appears particularly significant in vivo during tumor growth and less so in cells grown in tissue culture. The exact details of these phenomena remain to be further determined.

In this study, we focused on how circRNA17 alters the Enz resistance and cell invasion in CRPC cells via modulating the ARv7 expression. The circRNA17 has a high expression in hormone-sensitive PCa cells and a low expression in Enz-resistant PCa cells. Interestingly, compared with Enz-sensitive PCa cells, the cell invasion ability of Enz-resistant cells was also increased, suggesting a tight relationship between Enz resistance and tumor cell invasion and possibly PCa metastasis. The significance of ARv7 expression in the Enz resistance and cell invasion was underscored by their strict dependence on ARv7 expression. It is worth noting that the parental C4–2 cells, even though sensitive to Enz, already express ARv7 at the transcript level, yet the ARv7 protein expression is very low, likely due to the post-transcriptional regulation by miRNAs and in extension the circRNAs, among other potential mechanisms. This is also consistent with the previous finding that ARv7 mRNA was present in hormone-sensitive cells such as LNCaP cells^23^. These findings suggest that differential splicing of the AR gene precedes the expression of AR-Vs at the protein level, which contributes to the resistance to ADT treatment including Enz. Thus, the expression of ARv7 mRNA, even though positively correlated with resistance to Enz and arbiraterone, can still result in little expression of ARv7 protein, thus continued efficacy of ADT treatment. Prolonging this period through enhancement of post-transcriptional repression of ARv7 mRNA translation can be a booster of the existing ADT.

Furthermore, from the point of view of circRNA17/miR-181c-5p expression, Enz can impact Enz sensitivity immediately as Enz rapidly suppresses circRNA17 and miR-181c-5p expression, with a concomitant increase of ARv7, thus potentially increased resistance to Enz therapy. It is possible that the immediacy of the effect of Enz on ARv7 expression might be a reflection of the particular cell line and tissue culture condition, and may not be present in all patients or take a longer time. Although the change of ARv7 expression and its impact on Enz resistance is not completely reversed as a result of circRNA17 expression, it is indeed to be expected that circular RNA expression through regulation of miR-181c-5p is among several different mechanisms for the ARv7 expression and Enz resistance. More investigation is required to clarify the detailed mechanisms involved in the development of Enz resistance.

In conclusion, Enz could inhibit circRNA17 expression and decrease miR-181c-5p expression, thus induce ARv7 expression with a consequent resistance to Enz and increased cell invasion. Targeting this new signaling axis of circRNA17/miRNA-181c-5p/ARv7 thus may provide a novel therapeutic modality to enhance Enz efficacy while also to reduce its negative side effects such as acquired resistance to Enz and PCa metastasis.

## Electronic supplementary material

Fig.S1

Supplementary figure legends
