# Supplementary figures and images for: Preclinical study using circular RNA 17 and micro RNA 181c-5p to suppress the enzalutamide-resistant prostate cancer progression
Source: Cell Death Dis. 2019 Jan 15;10(2):37. doi: 10.1038/s41419-018-1048-1 (PMC6425037; doi:10.1038/s41419-018-1048-1)

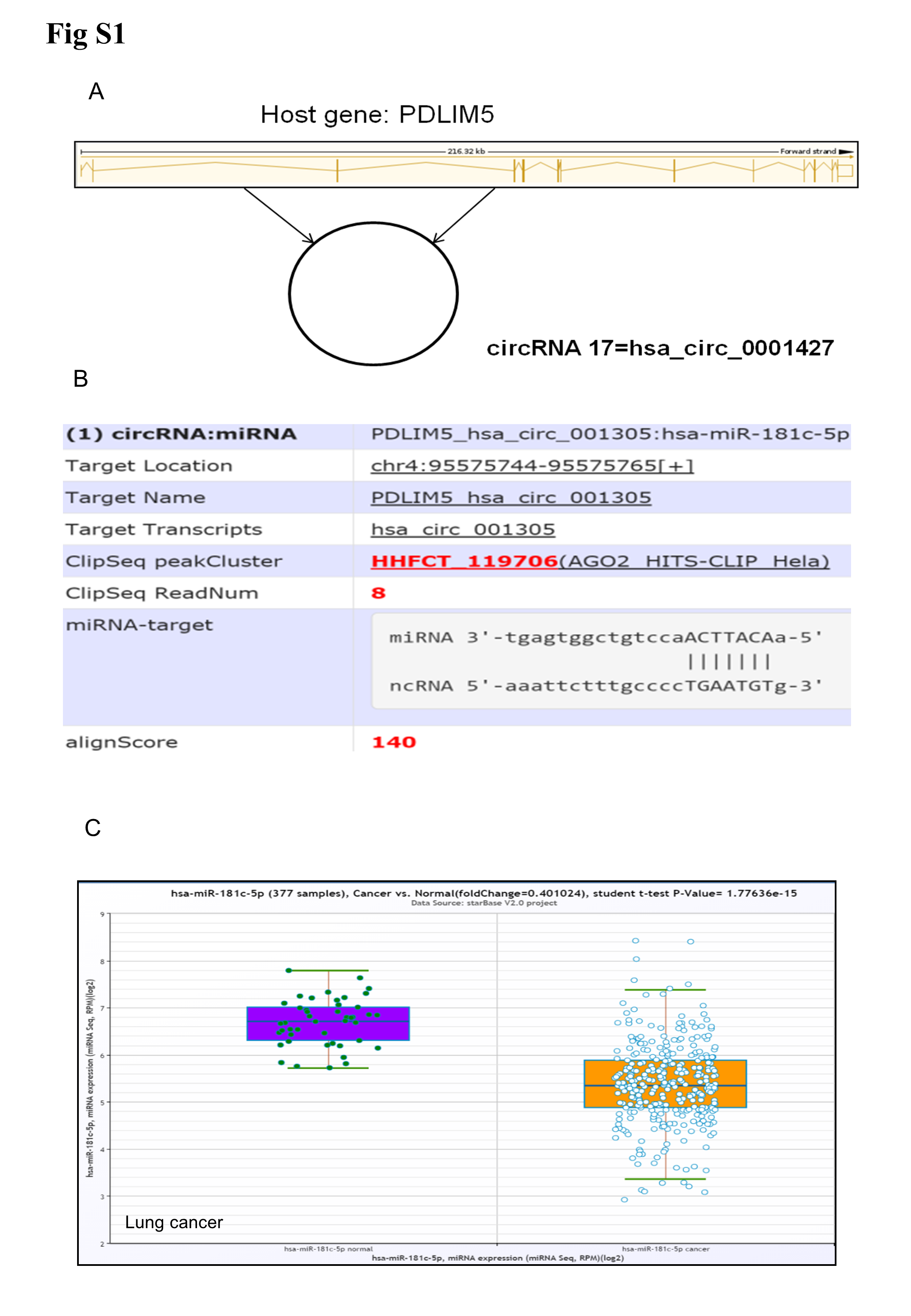

Supplement: Supplementary file 1 — Fig.S1 [file 41419_2018_1048_MOESM1_ESM.tif]
