# Supplementary material for: Preclinical study using circular RNA 17 and micro RNA 181c-5p to suppress the enzalutamide-resistant prostate cancer progression
Source: Cell Death Dis. 2019 Jan 15;10(2):37. doi: 10.1038/s41419-018-1048-1 (PMC6425037; doi:10.1038/s41419-018-1048-1)
Supplement: Supplementary file 2 — Supplementary figure legends [file 41419_2018_1048_MOESM2_ESM.docx]

**Figure S1: A.** The circRNA17 in relationship to its host gene PDLIM5. **B.** The binding site and sequence of circRNA17 with miR-181c-5p predicted through StarBase. **C.** Bioinformatic analysis (TCGA) shows that the miR-181c-5p expression is higher in lung cancer specimens than in normal specimens.
